# A high-throughput drug screening assay for anti-tau aggregation using split GFP and flow cytometry

**DOI:** 10.1038/s41598-025-21680-5

**Published:** 2025-10-29

**Authors:** Omnia M. H. Ibrahium, Taiwo A. Ademoye, Jessica S Fortin, Raluca Ostafe

**Affiliations:** 1 Purdue Institute of Inflammation, Immunology and Infectious Diseases, Purdue University, West Lafayette, IN 47907 USA; 2 Department of Basic Medical Sciences, College of Veterinary Medicine, Purdue University, West Lafayette, IN 47907 USA

**Keywords:** Biochemistry, Biological techniques, Biotechnology, Chemical biology, Drug discovery, Diseases, Neurology

## Abstract

Tau protein aggregation is a hallmark of neurodegenerative diseases, including Alzheimer’s disease, making the development of anti-aggregation therapeutics a critical area of research. Progress in drug discovery has been hindered by the lack of efficient screening methods that accurately reflect cellular conditions. We present a high-throughput cell-based assay utilizing split GFP technology to monitor tau aggregation in living cells. Our system employs suspension-adapted HEK293 cells co-transfected with tau proteins fused to complementary GFP fragments, producing fluorescent signals upon tau aggregation. Notably, our system demonstrates tau aggregation without external aggregation inducers, likely due to the enhanced protein expression in suspension-adapted cells. Validation with a known urea-based tau aggregation inhibitor showed dose-dependent reduction in fluorescence, corresponding to decreased tau aggregation. The assay’s flow cytometry compatibility enables rapid, quantitative analysis of large sample sets while allowing simultaneous assessment of compound efficacy and cytotoxicity. This method advances tau aggregation monitoring and drug discovery by providing a physiologically relevant platform for identifying novel anti-tau aggregation therapeutics.

**Supplementary Information:**

The online version contains supplementary material available at 10.1038/s41598-025-21680-5.

## Introduction

Tauopathies, including Alzheimer’s disease (AD), are a class of neurodegenerative disorders characterized by the pathological aggregation of the microtubule-associated protein Tau into neurofibrillary tangles (NFTs). These aggregates disrupt neuronal function, contributing to synaptic loss, neuronal death, and cognitive decline. Therapeutic interventions targeting Tau aggregation represent a promising strategy for mitigating disease progression. However, identifying effective compounds to prevent or reverse Tau aggregation requires robust and scalable screening platforms capable of capturing the complexity of Tau pathology in a physiologically relevant manner.

Contradictory to its pathological aggregation, Tau is an intrinsically disordered and highly soluble protein under normal physiological conditions^1,2^. Its transition to a pathogenic, aggregation-prone state involves a cascade of post-translational modifications (PTMs) such as hyperphosphorylation^3–6^, acetylation^7,8^, and truncation, as well as conformational changes within neurons^9,10^. Mutations in the Tau gene (*MAPT*)^11,12^, imbalances in expression of 3R and 4R isoforms^13–15^, and altered PTM profiles have been shown to tip the balance toward aggregation. These factors collectively lead to the formation of pathological Tau aggregates, including paired helical filaments (PHFs) and neurofibrillary tangles (NFTs), which are among the hallmarks of Tau-related neurodegeneration.

Multiple reviews addressing the cellular and pathological functions of tau and the events leading to disease are available^11,16,17^.

 In vitro studies of Tau aggregation are valuable for understanding the molecular structure of PHFs and have been widely used in drug screening efforts. However, progress has been limited by an incomplete understanding of Tau aggregation dynamics and the lack of reliable models that reflect the biological complexity of Tau pathology. Recombinant full length tau purified from *Escherichia coli* exhibits limited aggregation potential due to the absence of essential PTMs^18–20^. To address this limitation, researchers have employed truncated Tau isoforms, such as K18^21–23^ and K19^24,25^ (also known as repeat domains, RD) which contain the microtubule-binding region and aggregate more readily than full-length Tau^21,25^. Additionally, disease-relevant mutations, such as P301S^25^, P301L^26^ and ΔK280^27^, enhance β-sheet formation and have been incorporated into models to increase aggregation efficiency. The aggregation rate is further accelerated by the addition of artificial cofactors, which neutralize Tau’s lysine-rich positive charge. These cofactors fall into two main categories: polyanions (e.g., heparin, RNA^28^, polyglutamate^29^ and fatty acids or fatty acid-like molecules (e.g., arachidonic acid, docosahexaenoic acid). Polyanions, such as heparin^23,30^, are particularly effective at promoting the polymerization of truncated Tau fragments (e.g., K18 and K19), while arachidonic acid enhances the aggregation of full-length Tau more efficiently than heparin^22,31^.

Current approaches for detecting Tau aggregation in vitro rely heavily on fluorescence probes like thioflavin to monitor β-sheet formation and structural analyses using transmission electron microscopy^32–35^. While these methods provide valuable mechanistic insights, they are resource-intensive, time-consuming, and dependent on non-physiological starting materials. Although these approaches improve aggregation rates and provide insights into Tau pathology, they rely on artificial systems that lack the complexity of the native cellular environment. As such, these models may not fully replicate the physiological conditions of Tau aggregation and are often unsuitable for large-scale, high-throughput drug screening campaigns. Furthermore, they do not account for the effects of PTMs, co-factors, or intracellular interactions that are crucial to Tau aggregation.

 In vivo models, such as transgenic mice, offer a closer representation of human biology^36^ but are limited by ethical concerns, high costs, and a lack of scalability. These models are not practical for large-scale drug discovery efforts. As an alternative, cell-based assays provide a physiologically relevant and scalable platform for studying Tau aggregation. Mammalian cells can introduce post-translational modifications (PTMs), such as phosphorylation, acetylation, and glycosylation, that are absent in bacterial expression systems and more closely resemble those found in human proteins compared to yeast or insect cell systems. This makes mammalian cells a more suitable platform for studying drug effects on proteins and a potential intermediate model for investigating Tau aggregation dynamics. Previous studies with HEK cells have shown that glycosylation patterns of various human proteins are similar to native types^37^, and other studies using HEK cell lines suggest that Tau expressed in these systems exhibits phosphorylation patterns comparable to those found in neuronal contexts^38^. Additionally, cell-based systems enable simultaneous assessment of compound cell penetration—which is particularly important for Tau-targeting drugs, as Tau is an intracellular protein—along with cell viability and aggregation efficiency, making them well-suited for high-throughput applications.

There have been several cell-based assays for Tau aggregation described in the literature, as comprehensively reviewed by Lim et al.^20^. To provide context for the current work, we summarize these systems to highlight how our assay, presented in this paper, differs from and builds upon previous approaches. One of the earliest cell-based models for Tau aggregation was developed by Mandelkow’s group^39^. They generated a Tau-inducible cell line to address the toxicity associated with Tau aggregation in cells. Using an inducible system regulated by doxycycline, they overexpressed the truncated K18 Tau isoform in N2a neuroblastoma cells. While this approach demonstrated robust aggregation detectable by thioflavin S (ThS) staining, aggregation was observed only in the ΔK280 mutant and not in wild-type K18 Tau, even after nine days of expression. This highlights the challenges of inducing aggregation in physiologically relevant isoforms without introducing strong aggregation-prone mutations. Kuret’s and Lee’s groups showed full-length Tau-40 (2N4R Tau) aggregation in various cell lines only after adding small-molecule agonist of Tau aggregation (Congo red)^40^ or preformed Tau fibrils^41^. These models have significantly advanced the understanding of intracellular Tau aggregation and its associated cellular toxicity. However, most of these systems rely on secondary methods such as ThS staining or immunostaining against phosphorylated Tau to confirm aggregation. While effective, these approaches are labor-intensive and less suited for high-throughput screening applications.

To overcome these limitations, fluorescence-based systems have been developed, incorporating fluorescent tags such as GFP, CFP, or YFP fused to various Tau isoforms like Tau-40, K18, or K19, including mutated variants^42,43^. These models enable real-time monitoring of intracellular Tau expression in living cells without requiring secondary detection methods. However, these systems often encounter challenges in quantifying specific Tau–Tau interactions, as all Tau proteins in the assay are fluorescent, making it difficult to distinguish between aggregated and non-aggregated forms.

To address this, fluorescence resonance energy transfer (FRET) and bimolecular fluorescence complementation (BiFC) assays have been widely employed to study protein–protein interactions in general, as extensively reviewed elsewhere^44–46^, and have also been adapted for Tau–Tau interactions. For instance, Johnson’s group utilized FRET by tagging full-length Tau with CFP and YFP and expressing them in HEK293 cells, enabling the detection of GSK3β-induced aggregation via energy transfer when the proteins were in close proximity^47^. Similarly, Diamond’s group applied FRET to study the aggregation of the K18 version of Tau and explored its trans-cellular propagation. Their findings demonstrated that extracellular Tau fibrils could be taken up by neighboring cells, inducing intracellular aggregation and illustrating FRET’s utility in tracking both aggregation and propagation in living cells^48^.

In addition, they created a stable HEK293T cell line expressing the K18 Tau P301S mutant tagged with CFP and YFP, in which Tau aggregation can be induced by fibrils or other compounds. This cell line, available from ATCC (CRL-3275), can also be used in seeding assays that directly translate to human brain homogenates, further emphasizing the utility of FRET in studying Tau pathology^25^.

While FRET provides valuable insights into Tau aggregation, it has notable limitations, including the need for specialized equipment, low sensitivity, and potential interference from large fluorescent tags. Additionally, its efficiency depends on spectral overlap and dipole orientation of the fluorophores, which can complicate analysis. FRET also suffers from a limited dynamic range, weaker signals without amplification, and challenges in detecting stable protein–protein interactions^49^. These drawbacks highlight the need for complementary methods, such as BiFC, to overcome these limitations.

Johnson’s group utilized a split GFP complementation technique as a “turn-off” sensor to quantify Tau aggregation in cells. In this approach, full-length Tau was fused to a small GFP fragment (GFP11) and co-expressed with a larger GFP fragment (GFP1–10). Fluorescence was generated when Tau remained as monomers, but aggregation prevented GFP reconstitution^50^. While effective in demonstrating that mutants prone to aggregation do not produce fluorescence, this “turn-off” system is limited in its ability to monitor early aggregation events, such as soluble intermediates. Additionally, its application in screening campaigns for aggregation inhibitors or reversers is constrained by the extremely long half-life of the N-GFP and C-GFP interaction, estimated at 10 years^51^. The Venus-based BiFC system, using split Venus fragments (VN173 and VC155) to label Tau, addresses this limitation by acting as a “turn-on” sensor. Fluorescence activates only upon Tau aggregation, with minimal background under basal conditions. In HEK293 cells expressing full-length Tau, aggregation induced by phosphorylation-promoting compounds produced strong fluorescence, enabling detection of early aggregation events^52^.

Huttunen’s group employed a split GFP system with the 0N4R Tau isoform, although its functionality was not thoroughly investigated. Their study primarily focused on using the split GFP Tau system to examine the effects of conditioned media containing Tau on neighboring cells with respect to stress granules, rather than comprehensively validating its utility for detecting aggregation dynamics or high-throughput drug screening^53^. This highlights the need for more robust and systematically validated systems, such as the one presented in this study, to effectively study Tau aggregation and identify therapeutic modulators.

Building on Huttunen’s approach, we are using a similar split GFP construct with 0N4R Tau but specifically optimizing the system for drug screening applications. Our goal is to identify compounds capable of preventing Tau–Tau interactions in a scalable and efficient manner. Importantly, while most studies on cell-based Tau aggregation rely on microscopy-based methods, which are time-consuming and challenging to quantify accurately, we have adapted our system for flow cytometry, offering rapid and reliable quantification and high throughput.

One notable exception to the reliance on microscopy is a recent study by Allsop and Mudher’s group^54^, which used an ATCC cell line expressing K18 Tau to evaluate an aggregation inhibitor. However, their approach, like many others, uses adherent cells, which must be detached for flow cytometry analysis. Additionally, most publications report that Tau aggregation in cell-based systems requires the addition of seeds or other aggregation inducers. Even when truncated forms such as K18 mutants are used, aggregation without seeds is rarely observed. Allsop and Mudher’s work further noted that FRET signals in their system did not occur unless seeds were introduced.

In contrast, our system demonstrates that GFP fluorescence can be directly detected in suspension-adapted HEK293 cells co-transfected with split GFP constructs (GFP10 and GFP11) fused to 0N4R Tau. These suspension cells offer several advantages: they grow at higher densities, are less influenced by microtubule interactions, and produce greater amounts of protein. We hypothesize that this higher protein production enables the observation of Tau–Tau interactions in these cells, even without the need for adding aggregation inducers, a key difference compared to adherent HEK293 cells described in other studies. Furthermore, suspension cells are inherently better suited for flow cytometry-based applications, allowing for efficient, high-throughput screening of Tau aggregation inhibitors. This streamlined approach not only simplifies experimental workflows but also provides a robust and scalable platform for drug discovery.

## Results

Cells transfected with the Tau-split GFP construct exhibited a robust fluorescent signal, indicating the successful aggregation of Tau protein and the reassembly of split GFP fragments. FACS analysis revealed a significant reduction in the percentage of fluorescent cells in drug-treated samples compared to controls. The fluorescence intensity decreased in a dose-dependent manner, suggesting that the drug effectively inhibited Tau aggregation.

### Validation of the split GFP Tau system

**Fig. 1:**
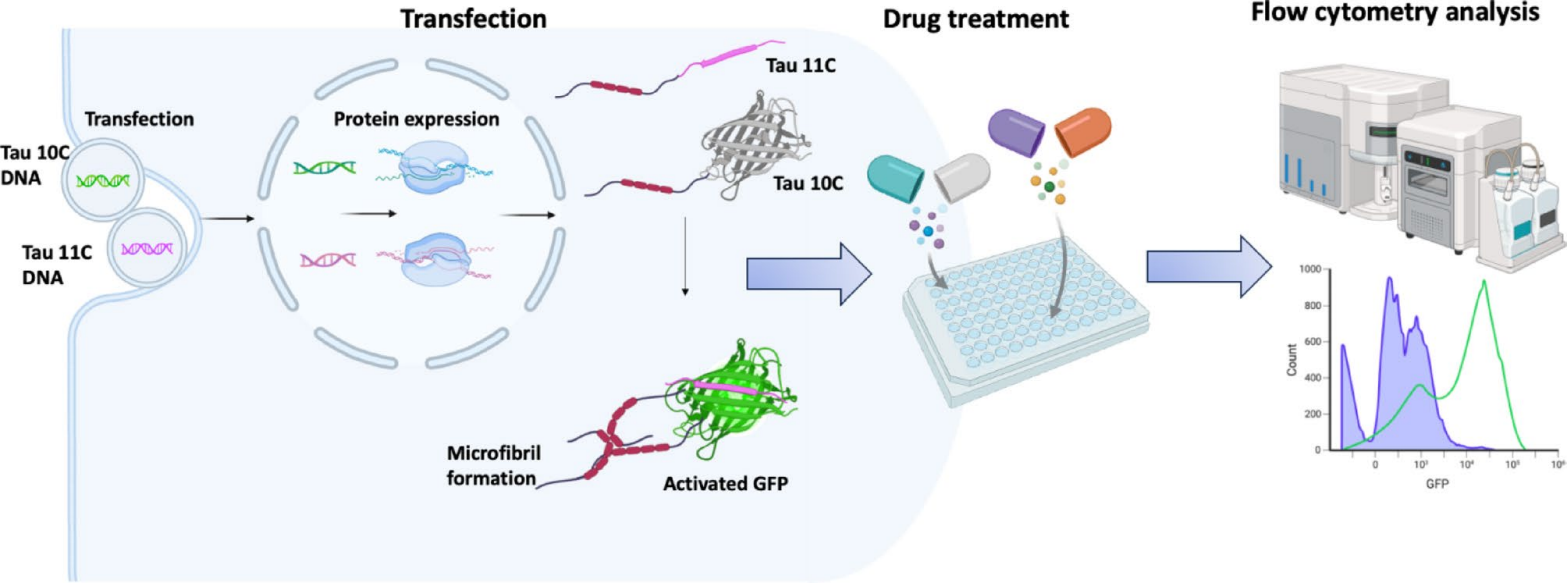
The assay employs split GFP fragments (GFP10 and GFP11) fused to 0N4R Tau to detect Tau aggregation in living cells. Co-transfection of Tau-GFP10 and Tau-GFP11 constructs leads to protein expression. Upon Tau aggregation, the GFP fragments reconstitute a functional GFP, producing a fluorescent signal. The assay can be scaled to microtiter plates for transfection, cell growth, and the addition of potential Tau aggregation inhibitors. The fluorescent signal is quantified using high-throughput methods such as flow cytometry, enabling efficient screening of candidate compounds that prevent or reduce Tau aggregation.

To validate the functionality of the split GFP-based system for detecting Tau aggregation, we co-transfected suspension-adapted HEK293 (Expi293) cells with plasmids encoding GFP10 and GFP11 fragments fused to the 0N4R Tau isoform. Upon aggregation of Tau, the GFP10 and GFP11 fragments reconstitute into a functional GFP, generating fluorescence that can be quantified using flow cytometry. The general principle of the assay and its application for drug screening is illustrated in Fig. 1. This system allows real-time monitoring of Tau aggregation in living cells without the need for secondary detection methods.

**Fig. 2:**
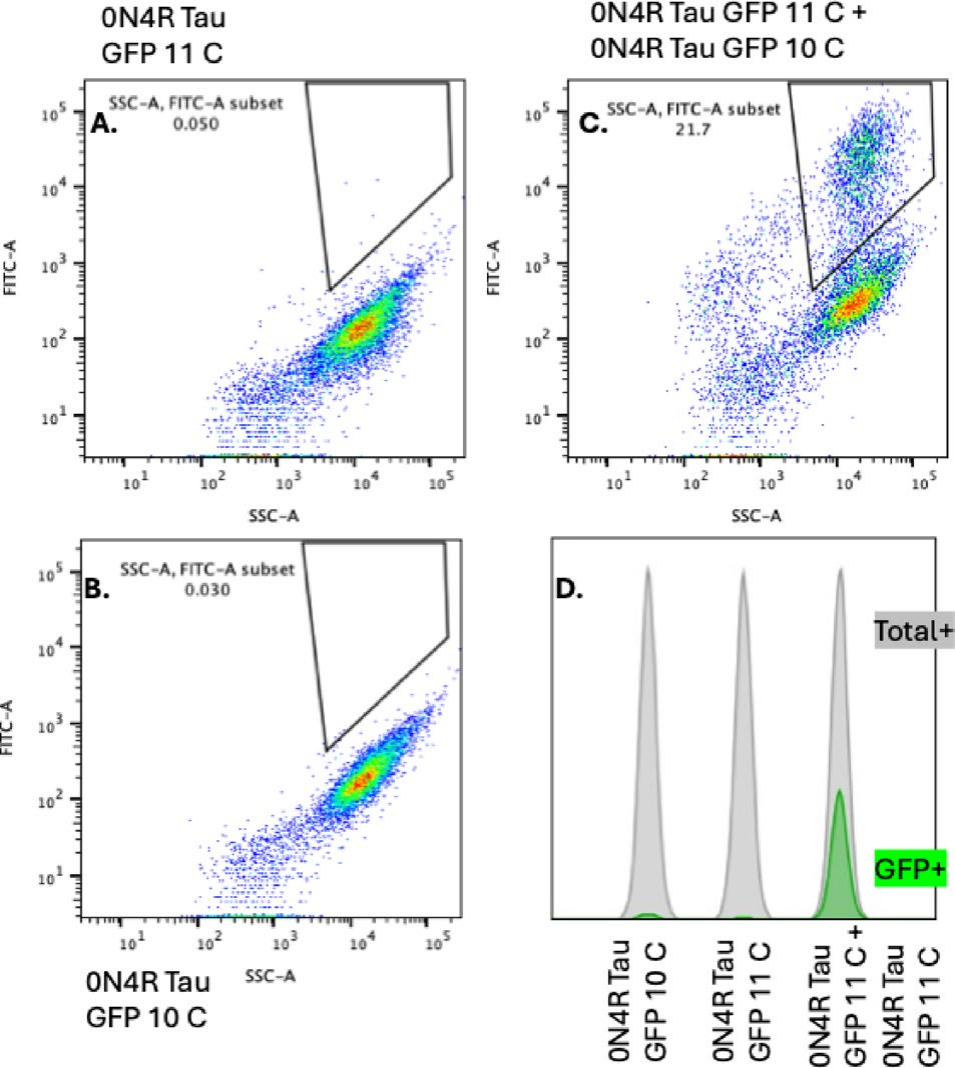
A and B: HEK293 cells transfected individually with GFP10-Tau (**A**) or GFP11-Tau (**B**) show negligible GFP fluorescence, confirming that neither split GFP fragment fluoresces on its own. (**C**) Co-transfection of cells with GFP10-Tau and GFP11-Tau results in a significant increase in GFP fluorescence, indicative of Tau–Tau interactions leading to GFP reconstitution. (**D**) Concatenated histogram comparing fluorescence profiles of individual transfectants (gray, Total+) and co-transfected cells (green, GFP+), highlighting the GFP-positive population resulting from Tau aggregation.

Flow cytometry was used to quantify fluorescence intensity as a measure of Tau aggregation. GFP fluorescence was robustly detected in co-transfected cells, with a clear separation between GFP-positive and GFP-negative populations. The fluorescence intensity correlated with the extent of aggregation, as evidenced by a shift in the fluorescence peak compared to negative controls, which included cells transfected with individual GFP fragments or empty plasmids (Fig. 2). These results confirm the ability of the split GFP system to detect Tau aggregation with high sensitivity and reproducibility. To further evaluate whether GFP fluorescence in this system is specifically driven by Tau–Tau interactions, we performed additional control experiments using alternative split-GFP construct combinations (Supplementary Figure S4). Co-expression of Tau-GFP11C with freely diffusible GFP10C yielded only weak fluorescence, suggesting that limited spontaneous complementation may occur when one fragment is unfused. In contrast, pairing Tau-GFP10C with Scarlet-GFP11C, a non-interacting fusion partner, resulted in negligible GFP signal. Strong GFP fluorescence was observed only when both split GFP fragments were fused to Tau, indicating that Tau–Tau interactions are required to bring the fragments into close proximity and enable reconstitution. These results confirm that fluorescence in our system reflects tau-dependent assembly rather than nonspecific fragment complementation.

### Time course analysis of split GFP expression to determine optimal expression levels

To determine the optimal time points for detecting Tau aggregation using the split GFP assay, we conducted a time-course experiment in Expi293 cells co-transfected with GFP10-Tau and GFP11-Tau constructs. GFP fluorescence was measured over multiple days post-transfection using flow cytometry.

**Fig. 3:**
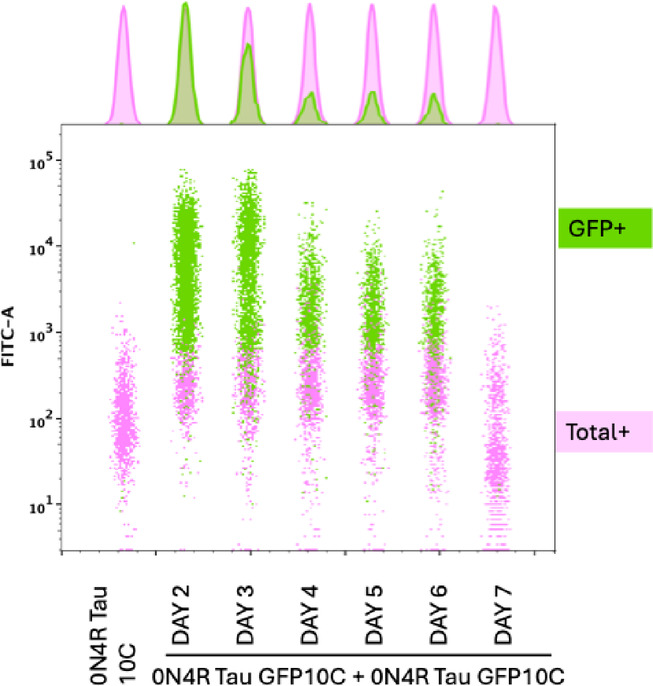
Time course of GFP expression in transfected cells using the split GFP Tau aggregation assay.

The concatenated histogram (Fig. 3) demonstrates that GFP fluorescence peaks at 48–72 h post-transfection, indicating maximum Tau aggregation and GFP reconstitution. A decline in fluorescence intensity was observed at later time points, likely due to reduced protein expression, cell death, or turnover of Tau aggregates.

These findings suggest that the optimal window for detecting Tau aggregation and assessing the effects of candidate drugs is between 48 and 72 h post-transfection. This time frame ensures robust GFP signal detection while maintaining cell viability and reproducibility for high-throughput drug screening.

### Drug candidate effect on Tau aggregation and cell viability

Having optimized conditions for the assay we tested a urea-based drug compound N-(3,4-dichlorophenyl)-N′-[2-(1 H-indol-3-yl)ethyl]urea) (Supplementary Figure S1) for the efficiency of inhibiting the aggregation of tau. The drug candidate had previously been tested against alpha synuclein anti-aggregation activity^35,58^ and in the context of this paper we have also proved the activity against different forms of Tau using the ThS assay (Supplementary Figure S2).

The effect of our test compound on tau aggregation and cell viability was evaluated using the split GFP system in Expi293 cells. Our analysis revealed several important findings.

**Fig. 4:**
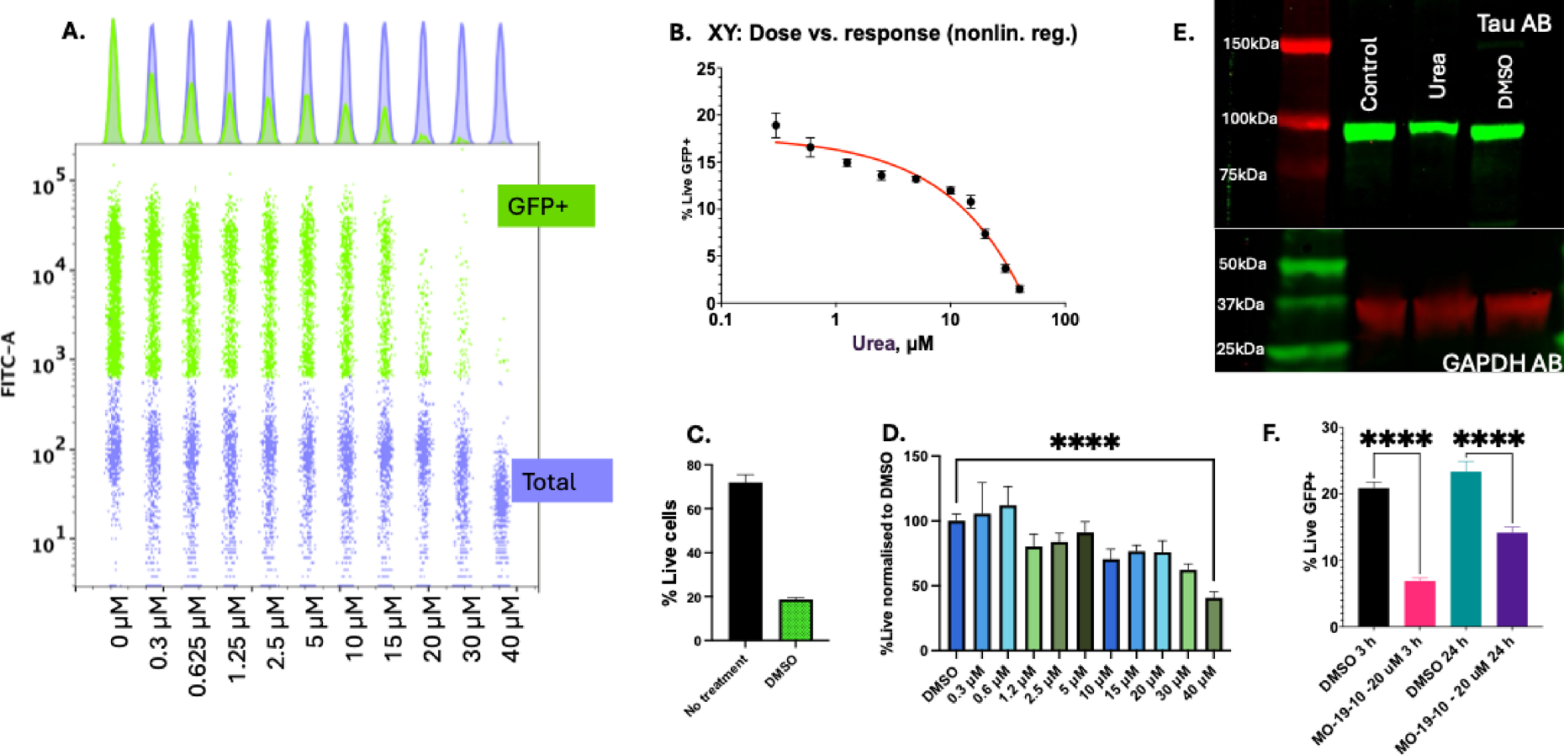
Analysis of drug candidate effect on tau aggregation and cell viability. (**A**) Concatenated histogram and scatter plot showing GFP fluorescence gated on live cells (GFP+, green) and total cell population (Total+, blue) across increasing concentrations of drug treatment in Expi293 cells co-transfected with GFP10-Tau and GFP11-Tau constructs. The 0 μM condition represents the DMSO vehicle control (0.1% final concentration), used in all treatment groups. (**B**) Nonlinear regression analysis of the dose-response curve using a four-parameter logistic model. The Hill slope was − 0.6554, indicating mild negative cooperativity in the inhibitory response. (**C**) Initial reduction in cell viability was observed with DMSO vehicle control compared to untreated cells. (**D**) Drug treatment showed significant cytotoxicity (*p* < 0.0001) only at concentrations above 30 μM when compared to DMSO-treated cells. (**E**) Western blot analysis revealed a reduction in the intensity of the ~108 kDa band corresponding to the Tau-GFP10C/Tau-GFP11C complex in urea based compund-treated samples, supporting the observed inhibition of Tau aggregation. with either DMSO, urea compound or no treatment. GAPDH was used as a loading control. Full-length, uncropped blots corresponding to this figure are provided in Tau WB Images.pptx Supplementary Files. (**F**) Comparison of drug addition time points post-transfection. Drug compounds added at 3 h post-transfection demonstrated a more pronounced inhibition of Tau aggregation compared to those added at 24 h post-transfection.

The drug candidate demonstrated clear dose-dependent inhibition of tau aggregation, as evidenced by the progressive reduction in GFP-positive cells across increasing concentrations (Fig. 4A). The dose-response curve in (Fig. 4B). was generated using nonlinear regression with a four-parameter logistic model (variable slope). The Hill slope was calculated to be − 0.6554, indicating a gradual, concentration-dependent inhibitory effect on Tau aggregation. This shallow slope reflects a broad and progressive response, rather than steep or cooperative behavior. The IC_50_ value could not be reliably constrained due to the flatness of the curve and overlapping cytotoxicity at higher concentrations. This trend may reflect a complex inhibition mechanism, such as partial target saturation, multiple non-equivalent binding sites on Tau, or indirect effects where the compound interferes with early aggregation steps but not mature aggregates. Importantly, we do not suggest that this assay is suitable for precise IC_50_ determination, given the confounding variables present (e.g., cytotoxicity, protein overexpression, and potential off-target effects). Rather, our intention is to demonstrate that Tau-GFP oligomer formation is clearly influenced by compound concentration, validating the system as a tool for detecting relative anti-aggregation activity.

However, our viability analysis revealed important considerations regarding the assay conditions. The addition of DMSO as a vehicle control resulted in a baseline reduction in cell viability compared to untreated cells (Fig. 4C). While 0.1% DMSO reduced overall cell viability compared to untreated cells (Fig. 4C), all samples were vehicle-matched. Moreover, both GFP signal and viability were quantified by gating only on GFP + viable cells, ensuring that compound effects on Tau aggregation are evaluated independently of general DMSO-induced cytotoxicity.

When evaluating the drug’s effect on cell viability against DMSO-treated controls, significant cytotoxicity was only observed at concentrations exceeding 30 µM (Fig. 4D). This suggests that the compounds anti-aggregation effects at lower concentrations are not primarily due to cellular toxicity.

These findings indicate that while our test compound effectively inhibits tau aggregation, careful consideration of both vehicle effects and concentration-dependent toxicity is essential for accurate interpretation of drug screening results. The optimal therapeutic window appears to be below 30 µM, where tau aggregation is inhibited without significant cytotoxicity beyond vehicle effects.

The results of the GFP fluorescence assays were further supported by Western blot analysis, which revealed a decrease in the high-molecular-weight band (~ 108 kDa) corresponding to aggregated GFP10C–Tau and GFP11C–Tau complexes (Fig. 4E). For reference, the molecular weights of the individual constructs are ~ 64.7 kDa for GFP10C–Tau and ~ 43.8 kDa for GFP11C–Tau, while the 0N4R Tau monomer alone is ~ 40 kDa. Additional, even higher molecular weight Tau species were observed and are presented in Supplementary Figure S3 (panels A–D). These include bands retained in the stacking gel or faint high-MW smears visible only after extended blotting and when probed with oligomer-selective antibodies (TOMA-1, T22). These findings suggest that more extensive Tau aggregation occurs in the cells, beyond the dimeric GFP10C–Tau / GFP11C–Tau complex. However, since the split-GFP fluorescence assay used in this study only reports on interactions between two Tau molecules (enabling GFP reconstitution), we focused the main data on the ~ 108 kDa species, which directly corresponds to the fluorescence signal quantified by flow cytometry.

### Cell-type dependent Tau aggregation: enhanced detection in suspension-adapted HEK293 cells

**Fig. 5:**
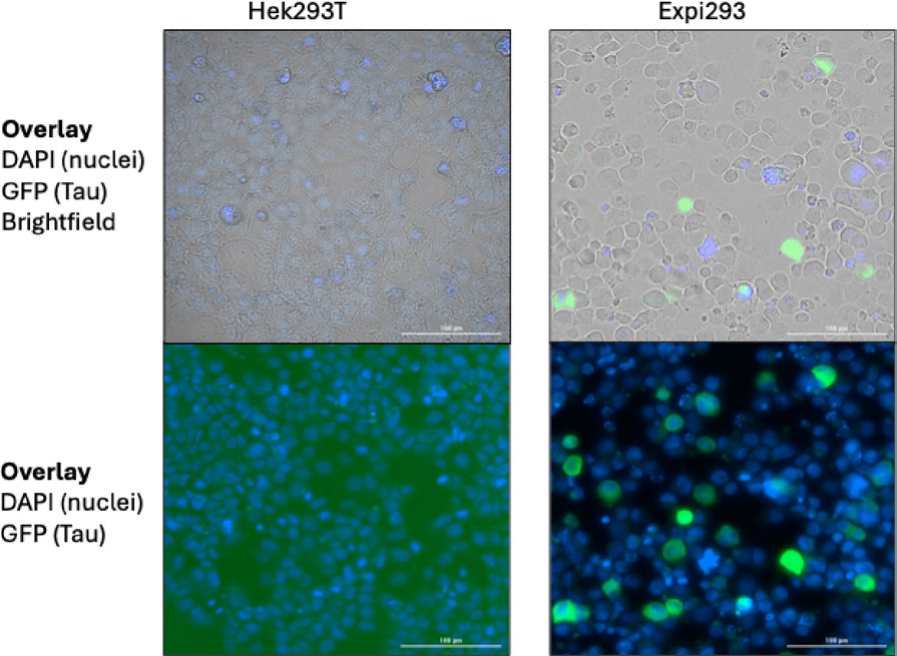
Fluorescent microscopy images of HEK293T adherent cells and suspension-adapted Expi(HEK)293 cells co-transfected with GFP10-Tau and GFP11-Tau constructs. Scale bars indicate 100 μm.

Previous reports have consistently indicated that Tau aggregation is not typically observed in cell-based models without the addition of aggregation inducers, such as preformed tau seeds, even when using sensitive detection systems like FRET or split GFP. As these studies predominantly employed HEK293T adherent cells, we investigated whether cell type influences Tau aggregation by comparing our 0N4R split GFP constructs in both adherent and suspension-adapted HEK293 cells (Expi293) (Fig. 5).

We transfected both cell types with identical concentrations of the split GFP-Tau constructs and monitored fluorescence after 48 h using fluorescence microscopy. Consistent with prior studies, HEK293T adherent cells showed no detectable fluorescence signal above background levels. In contrast, Expi293 cells exhibited robust fluorescence.

## Discussions and conclusions

Our study demonstrates the development and validation of a sensitive split GFP-based system for monitoring Tau aggregation in Expi293 live cells. This assay successfully detected both spontaneous Tau aggregation and its inhibition by small molecule compounds, as confirmed through multiple experimental approaches including flow cytometry and Western blot analysis. The robust correlation between fluorescence signal reduction and decreased Tau aggregation validates this system’s utility for high-throughput screening of anti-aggregation compounds.

This method presents several novel distinct advantages over traditional in vitro aggregation assays. First, it enables real-time detection of Tau aggregation within a cellular environment, providing physiologically relevant insights into aggregation dynamics. Second, the high sensitivity of the split GFP system allows detection of early-stage aggregation events that might be missed by conventional methods. Third, its compatibility with flow cytometry facilitates rapid, quantitative analysis of large sample sets, making it particularly suitable for high throughput drug screening applications. Most current Tau aggregation assays rely on bulk fluorescence methods, such as Thioflavin T (ThT) binding^23,30,31^, where recombinant Tau is induced to aggregate (typically by polyanions like heparin) and aggregation kinetics are monitored over 24–48 h in microplate readers, which remain occupied throughout the assay, greatly increasing cost and decreasing throughput. Imaging-based techniques, such as FRET biosensors or immunofluorescence, also require seeded aggregation and prolonged imaging sessions^47,48,52^, often followed by complex image processing. While high-content imaging systems can automate these workflows, they remain time-intensive and frequently require cell fixation and nuclear staining for autofocus, increasing error rates and limiting throughput. In contrast, flow cytometry—particularly when paired with plate-based autosamplers—enables rapid, high-throughput single-cell analysis by interrogating each cell in the same optical plane. Data acquisition is highly efficient (10–30 s per well, or under 1 h for a full 96-well plate, and depending on the cytometer and cell concentration, a full plate can be analyzed in as little as 15 min). In the context of this assay, we can analyze live, unfixed, and unstained cells directly from the culture medium without additional washing steps, which further reduces error and improves throughput. Although flow cytometry is specialized equipment, platforms such as the Attune NxT (Thermo Fisher), BD High Throughput Sampler, Sartorius iQue 3/5, and IntelliCyt iQue Screener Plus are increasingly accessible in core facilities. Even without autosamplers, manual acquisition rates of approximately 100 samples per hour are achievable. Furthermore, flow cytometry has already been successfully applied in high-throughput drug screening campaigns across diverse targets, as reviewed elsewhere^55,56^, and has also recently been used in Tau-related screening approaches, albeit with seed-based models^54^.

A notable finding from our study was the observation of spontaneous Tau aggregation in suspension-adapted HEK293 cells without the need for aggregation inducers. This contrasts with previous reports using similar Tau constructs in adherent cell lines, where aggregation typically required the addition of preformed seeds or other inducing agents. This unexpected result highlights the critical role of cellular context in Tau aggregation studies.

We propose several mechanisms that might explain this cell type-dependent behavior. The primary factor likely relates to the fundamental differences in protein expression profiles between adherent and suspension-adapted cells. Suspension-adapted HEK293 cells, engineered for optimal protein production in biomanufacturing applications, typically demonstrate substantially higher protein expression levels than their adherent counterparts. This enhanced expression stems from multiple cellular adaptations, including the ability to grow at higher cell densities and selection for robust protein production machinery during the adaptation process. Given the concentration-dependent nature of protein aggregation kinetics, these elevated expression levels could significantly increase the probability of Tau–Tau interactions, facilitating split GFP complementation and the observed fluorescence.

Additionally, differences in cellular architecture may play a crucial role. Adherent cells maintain a rigid cytoskeletal structure due to surface attachment, potentially constraining protein mobility and interactions. The mechanical forces generated through surface attachment might also influence protein conformations in ways that inhibit Tau–Tau interactions. In contrast, suspension cells’ more flexible architecture might provide an environment more conducive to protein–protein interactions.

Future studies could further optimize this system by incorporating different Tau variants, particularly those associated with specific tauopathies. Integration of disease-specific mutations could provide valuable insights into mutation-specific aggregation mechanisms and facilitate the development of targeted therapeutics. Additionally, investigation of the precise mechanisms underlying the cell type-dependent differences in Tau aggregation could reveal new aspects of cellular regulation of protein aggregation.

A limitation of our current study is that we have not directly confirmed whether the observed Tau aggregates are filamentous, sarkosyl-insoluble, or share structural characteristics with pathological aggregates seen in tauopathies. Additional analyses, such as electron microscopy, sarkosyl fractionation, or Thioflavin staining, will be conducted in future studies to further characterize these aggregates. It is important to note that, in this assay, the signal reflects GFP complementation between Tau-GFP10C and Tau-GFP11C fusion proteins, which reports on Tau–Tau interactions but does not necessarily provide structural information beyond the dimeric or oligomeric state. Our method is designed as a high-throughput screening platform for identifying potential anti-aggregation compounds. As with any screening assay, further validation of promising compounds in more physiologically relevant models, including animal studies and clinical trials, will be necessary. Nevertheless, we believe this approach offers an efficient and robust tool for the rapid pre-selection of candidate drugs with anti-Tau aggregation potential.

Another important consideration is that the reconstituted GFP signal in our split GFP assay is highly stable and does not readily dissociate once formed. Nonetheless, our data (Fig. 4F) indicate that when the compound is added 24 h post-transfection—after GFP-Tau complexes have already formed—a slight reduction in GFP fluorescence is still detectable. This suggests that while the system is primarily suited for screening compounds that prevent Tau aggregation, it may still offer limited insight into agents with partial disaggregation activity. Therapeutic candidates identified with this assay should be further validated using complementary methods capable of assessing aggregate clearance.

The observed DMSO sensitivity likely reflects a combination of solvent cytotoxicity, transfection-induced membrane stress, and prolonged exposure (48–72 h), which is not typical for standard cell culture protocols where DMSO is quickly removed (e.g., post-thaw). Despite this, our data analysis focuses exclusively on GFP + viable cells, ensuring the specificity of the observed drug effects.

Another factor to consider is the apparent sensitivity of Expi293 suspension cells to the 0.1% DMSO used for drug solubilization. This sensitivity likely results from the combined effects of prolonged DMSO exposure (48–72 h), the presence of transfection reagents, and suspension/shaking culture conditions, all of which can increase membrane stress. Nevertheless, all experimental samples, including controls, were vehicle-matched, ensuring that any DMSO effects were normalized across conditions. Furthermore, both GFP signal and cell viability were quantified exclusively by gating on GFP + viable cells, minimizing potential confounding effects from DMSO-induced cell loss.

This work has significant implications for both basic research and drug discovery efforts in the field of tauopathies. The combination of spontaneous aggregation in suspension cells and compatibility with high-throughput screening methods makes this system particularly valuable for therapeutic development. Future applications could include screening large compound libraries for novel anti-aggregation agents and investigating cellular factors that modulate Tau aggregation.

## Materials and methods

### Cell culture and maintenance

Expi293™ cells (Thermo Fisher Scientific, Catalog #A14527, Waltham, MA, USA) were cultured in Expi293™ Expression Medium (Thermo Fisher Scientific) at 37 °C with 8% CO_2_ in a humidified incubator with orbital shaking at 125 rpm. Cell cultures were maintained between 0.3 × 10^6^ and 5 × 10^6^ cells/mL with greater than 95% viability through passaging every 3–4 days. Cell density and viability were monitored using a Countess II FL Automated Cell Counter (Thermo Fisher Scientific).

For adherent cell culture, HEK293T cells (ATCC^®^ Manassas, VA, USA # CRL-3216™) were maintained in DMEM (Corning, Catalog #10-013-CV.) supplemented with 10% fetal bovine serum (Corning, Catalog #35-010-CV) and 1% penicillin-streptomycin (100 U/mL penicillin, 100 µg/mL streptomycin) (Thermo Fisher Scientific, Catalog #15140122) at 37 °C in a humidified atmosphere containing 5% CO_2_. Cells were passaged at approximately 80% confluency using 0.25% trypsin-EDTA solution (Thermo Fisher Scientific, Catalog #25200056).

### Plasmid construction and Preparation

The split GFP-Tau fusion constructs pmGFP11C-Tau and pmGFP10C-Tau (Addgene plasmids #71434 and #71433, gifts from Henri Huttunen, University of Helsinki, Helsinki, Finland)^53^ encode the 0N4R isoform of Tau protein fused to complementary fragments of GFP. The pcDNA3.1-GFP(1–10) was obtained from Addgene # 70219^57^. The pcDNA3.1-Scarlet GFP 11 was ordered as a synthetic construct from GenScript. Plasmids were transformed into XL10-Gold ultracompetent *E. coli* cells (Agilent Technologies, Catalog #200314, Santa Clara, CA, USA) and grown on LB agar plates containing kanamycin (50 µg/mL) for pmGFP11C-Tau and ampicillin (100 µg/mL) for pmGFP10C-Tau, pcDNA3.1-GFP(1–10) and pcDNA3.1-Scarlet GFP 11 selection. Single colonies were cultured in LB broth containing respective antibiotics at 37 °C with shaking at 250 rpm for 16–18 h. Plasmids were purified using PureLink HiPure Plasmid Maxiprep Kit (Thermo Fisher Scientific, Catalog #K210006) following manufacturer’s instructions. DNA concentration and purity (A260/A280 ratio) were assessed using BioTek Neo2 Microplate Spectrophotometer (BioTek Instruments, Winooski, VT, USA) with Take3 micro-volume plates.

### Transfection procedures

Expi293™ cells were seeded at 3 × 10^6^ cells/mL the day before transfection and adjusted to 6 × 10^6^ cells/mL the next day, and co-transfected with pmGFP11C-Tau and pmGFP10C-Tau plasmids (1:1 ratio, 1 µg total DNA/mL) using ExpiFectamine™ 293 Transfection Kit (Thermo Fisher Scientific, Catalog #A14524). ExpiFectamine™ 293 Transfection Enhancer was added 16–24 h post-transfection. Transfections were performed in 5 mL cultures using 50 mL Corning^®^ conical mini bioreactor tubes (Corning, Catalog # 431720) maintained in a shaking incubator at 200 rpm. For high-throughput screening, transfections were carried out in 96-well plates sealed with gas-permeable adhesive seals (Thermo Fisher Scientific, Catalog # 249720) and shaken at 500 rpm. The cultures were maintained at 37 °C with 8% CO_2_ in a humidified incubator.

For adherent HEK293T cells, transfections were performed at 70–80% confluency in 96-well plates using jetOPTIMUS^®^ DNA Transfection Reagent (Polyplus-transfection Inc., Catalog #117-15, New York, NY, USA) at a DNA : reagent ratio of 1:1. The cultures were maintained at 37 °C with 8% CO_2_ in a humidified incubator.

### Drug treatment and dose-response studies

The aggregation inhibitor, referred to in this paper as the urea compound (MO-19-10, Compound 12, as described in^58^, N-(3,4-dichlorophenyl)-N′-[2-(1 H-indol-3-yl)ethyl]urea), was dissolved in DMSO to prepare a 40 mM stock solution. Cells were treated either three or twenty-four hours post-transfection with varying concentrations of the compound (0.3–40 µM) or a vehicle control (0.1% DMSO final concentration) for 48 h. All conditions were performed in biological triplicates.

### Flow cytometry analysis

Flow cytometry analysis was performed 72 h post-transfection. Cells were diluted in PBS at 1 × 10^6^ cells/mL and analyzed using a BD LSRFortessa analyzer (BD Biosciences, San Jose, CA, USA) using the 488 nm laser for GFP excitation (530/30 nm filter) and 561 nm laser for ethidium homodimer-1 detection (610/20 nm filter). Ethidium homodimer-1 (Thermo Fisher Scientific, Catalog #E1169) was added to assess cell viability. Forward scatter, side scatter, and fluorescence signals were recorded for a minimum of 10,000 events per sample using BD FACSDiva software (version 9.0).

For high-throughput analysis directly from microtiter plates, samples were analyzed using an Attune NxT Flow Cytometer (Thermo Fisher Scientific) equipped with an autosampler. Data acquisition parameters were maintained consistent with the standard analysis protocol.

### Microscopy analysis

Fluorescence microscopy was performed using a BioTek Cytation™ 5 Cell Imaging Multi-Mode Reader (Agilent Technologies, formerly BioTek Instruments, Santa Clara, CA, USA). For nuclear visualization, cells were stained with Hoechst 33342 (Thermo Fisher Scientific, Catalog #H3570) at a final concentration of 1 µg/mL for 15 min prior to imaging. GFP fluorescence was detected using a GFP filter cube (Ex: 469/35 nm, Em: 525/39 nm), while Hoechst fluorescence was detected using a DAPI filter cube (Ex: 377/50 nm, Em: 447/60 nm). Images were acquired using a 20× objective with Gen5™ Image + software (version 3.13). For comparison between adherent and suspension cells, both brightfield and fluorescence images were captured using identical exposure settings and analyzed using the same software parameters.

### Western blot analysis

Cells were harvested and lysed in RIPA buffer (Thermo Fisher Scientific, Catalog #89900) supplemented with Halt™ Protease and Phosphatase Inhibitor Cocktail (Thermo Fisher Scientific, Catalog #78440). Total protein concentration was determined using the Pierce™ BCA Rapid Gold Protein Assay Kit (Thermo Fisher Scientific, Catalog #A53225). Protein samples (50 µg per lane) were separated on 7.5% Bio-Rad TGX™ Stain-Free™ precast gels (Bio-Rad Laboratories, Catalog #4568024) and transferred to nitrocellulose membranes using the Bio-Rad Trans-Blot^®^ Turbo™ Transfer System with the high molecular weight protein program according to manufacturer’s instructions.

Membranes were blocked with 5% non-fat dry milk in PBS-T (PBS containing 0.1% Tween-20) for 1 h at room temperature. For Tau protein detection, membranes were incubated with mouse-anti-Tau primary antibody (Proteintech, Catalog #66499-1-Ig 1:10,000) followed by IRDye^®^ 800CW Goat anti-Mouse secondary antibody (LI-COR Biosciences). GAPDH was detected using mouse anti-GAPDH primary antibody (Proteintech, Catalog #PT 60004-1-Ig, 1:10,000) followed by IRDye^®^ 800CW Goat anti-Mouse secondary antibody (LI-COR Biosciences). Each antibody incubation step was performed for 1 h followed by three 10 min washes with PBS-T. Protein bands were visualized using the LI-COR Odyssey^®^ Imaging System.

### Thioflavin S (ThS) fluorescence assay

For the Thioflavin S (ThS) assay, a black 384-well flat-bottom microplate (Brand, ref 784076, medium binding) was employed. PBS (pH 7.4) from Gibco (catalog number 10010-023), treated with chelex beads (Biosciences, BTNM-0024), was added to each well to reach a final volume of 10 µL. The 0N3R, 0N4R, 2N3R, 2N4R tau peptide (obtained from rPeptide) was then introduced to achieve a final concentration of 12 µM, followed by the addition of DTT to 5 mM. The test compound was added to each well at a final concentration of 100 µM, and heparin was included at 150 µM. Arachidonic acid was then added to the mixture to reach a final concentration of 0.092 µg/mL (A0781, Fischer TCI). Thioflavin S (ThS) was incorporated at a final concentration of 40 µM to monitor fibril formation kinetics. Fluorescence intensity was recorded every 5 min, with a 30-s agitation step preceding each measurement.

#### Data analysis and statistics

Flow cytometry data were analyzed using FlowJo software (version 10.10, BD Biosciences, San Jose, CA, USA). Live cells were gated based on forward and side scatter properties and ethidium homodimer-1 exclusion. GFP-positive populations were determined using single-transfected cells (either Tau-GFP10C or Tau-GFP11C) as negative controls. The percentage of GFP-positive cells were calculated for each sample. Statistical analyses were performed using GraphPad Prism (version 10.4.1, GraphPad Software, San Diego, CA, USA). Statistical significance between treatment groups was determined using one-way ANOVA followed by Tukey’s multiple comparisons test, with *p* values < 0.05 considered statistically significant. Data are presented as mean ± standard deviation from at least three independent experiments.

Figure schematics were created using BioRender.com.

The authors used ChatGPT (OpenAI, San Francisco, CA, USA) for grammar and language correction assistance during manuscript preparation. The tool was not used for generating scientific content, data analysis, or interpretation.

## Supplementary Information

Below is the link to the electronic supplementary material.

Supplementary Material 1

Supplementary Material 2

## Data Availability

The datasets used and/or analyzed during the current study are available from the corresponding author on reasonable request.
