# Supplementary material for: A high-throughput drug screening assay for anti-tau aggregation using split GFP and flow cytometry
Source: Sci Rep. 2025 Oct 29;15:37866. doi: 10.1038/s41598-025-21680-5 (PMC12572213; doi:10.1038/s41598-025-21680-5)
Supplement: Supplementary file 1 — Supplementary Material 1 [file 41598_2025_21680_MOESM1_ESM.docx]

**Title:**

A High-Throughput Drug Screening Assay for Anti-Tau Aggregation Using Split GFP and Flow Cytometry

**Authors**

Omnia M. H. Ibrahium^1,^; Taiwo A. Ademoye^2^; Jessica S Fortin^2^; Raluca Ostafe^1^*

1. Purdue Institute of Inflammation, Immunology and Infectious Diseases, Purdue University, West Lafayette, IN 47907, USA
2. Department of Basic Medical Sciences, College of Veterinary Medicine, Purdue University, West Lafayette, Indiana 47907, United States

Supplementary data

Figure S 1 MO-19-10, N-(3,4-dichlorophenyl)-N′-[2-(1H-indol-3-yl)ethyl]urea, chemical formula


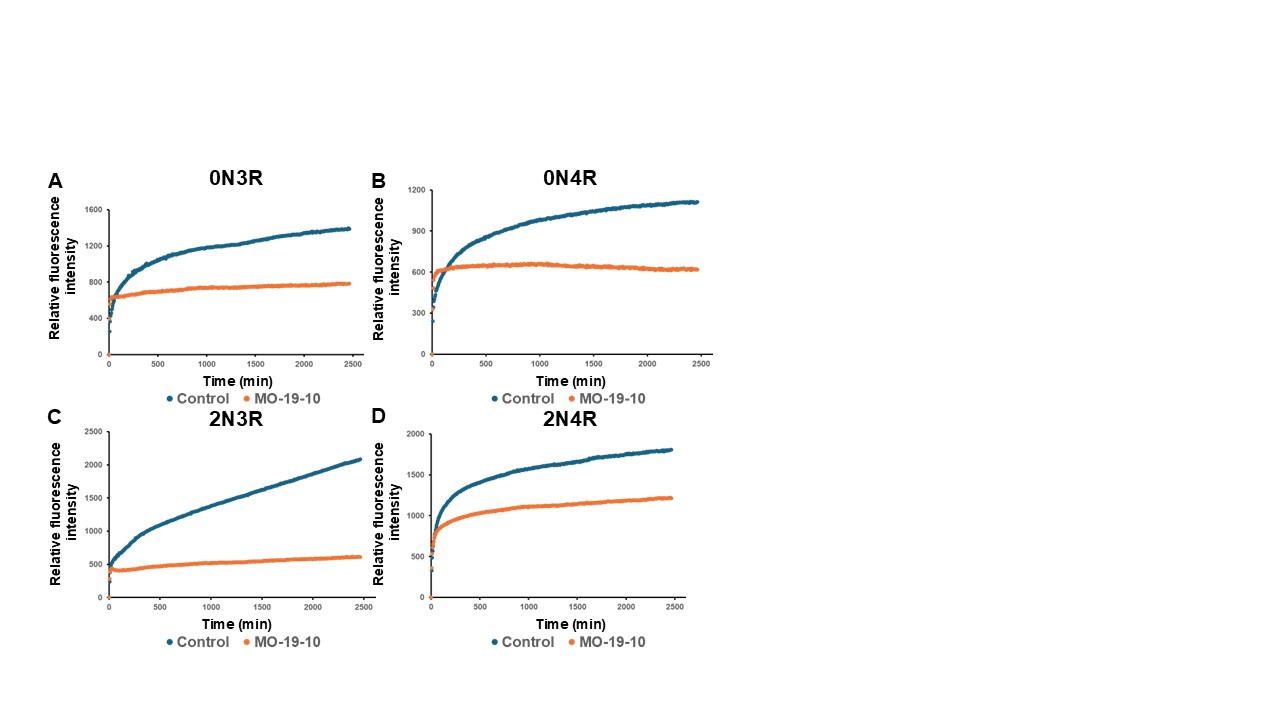


Figure S 2 MO-19-10, a urea-based compound, reduced the aggregation of tau 2N3R isoform by about 75% and other isoforms by less than 50%. The ThS fluorescence curves are indicative of the aggregation kinetics of tau isoform (A) 0N3R, (B) 0N4R, (C) 2N3R, and (D) 2N4R at 12 μM in PBS-treated with chelex beads. The solution was supplemented with 150 μM heparin, 5 mM dithiothreitol (DTT), 40 μM ThS, and 10 mg/mL arachidonic acid in order to induce the aggregation. The control condition contained 0.25% DMSO, while the urea compound was tested at a concentration of 100 μM. The data represented by each curve are an average of three independent replicates.

Figure S 3. Additional Western blot analysis showing high-molecular-weight Tau species and oligomers.

(A) SDS-PAGE gel stained for total protein, illustrating high-molecular-weight material retained near the stacking region in DMSO-treated samples, which is reduced in urea-treated samples.

(B) Western blot using a monoclonal anti-Tau antibody under extended transfer conditions; blot probed with the monoclonal anti-Tau antibody (proteintech) reveals faint higher-molecular-weight bands (>100 kDa) in DMSO-treated samples, consistent with SDS-resistant Tau oligomers, which decrease following urea treatment.

(C) Western blots probed with TOMA-1 (Sigma, MABN819) and T22 (Sigma, ABN454) antibodies, which are detetct Tau oligomers, confirm the presence of oligomeric Tau species in untreated samples and their reduction in urea-treated samples. These results further validate the anti-aggregation effects observed in our flow cytometry assay.

*Full-length, uncropped blots corresponding to this figure are provided in Tau WB Images.pptx Supplementary Files.*

Figure S 4 Control experiments assessing the dependence of GFP signal on Tau-Tau interactions.

Flow cytometry histograms showing GFP fluorescence in Expi293 cells transfected with various combinations of constructs containing split GFP fragments (GFP10C or GFP11C), with or without Tau fusion partners. GFP signal was observed only when both fragments were present and fused to Tau (Tau-GFP10C + Tau-GFP11C), suggesting that Tau-Tau interactions are required for efficient GFP reconstitution. Co-expression of Tau-GFP11C with unfused GFP10C resulted in weaker fluorescence, indicating partial spontaneous complementation. Conversely, co-expression of Tau-GFP10C with Scarlet-GFP11C, a fusion lacking Tau interaction capability, showed minimal fluorescence. These results support the conclusion that aggregation-driven proximity of Tau fusion constructs is necessary for robust GFP complementation in this system.
