# Supplementary material for: A high-throughput drug screening assay for anti-tau aggregation using split GFP and flow cytometry
Source: Sci Rep. 2025 Oct 29;15:37866. doi: 10.1038/s41598-025-21680-5 (PMC12572213; doi:10.1038/s41598-025-21680-5)
Supplement: Supplementary file 2 — Supplementary Material 2 [file 41598_2025_21680_MOESM2_ESM.pptx]

## Slide 1
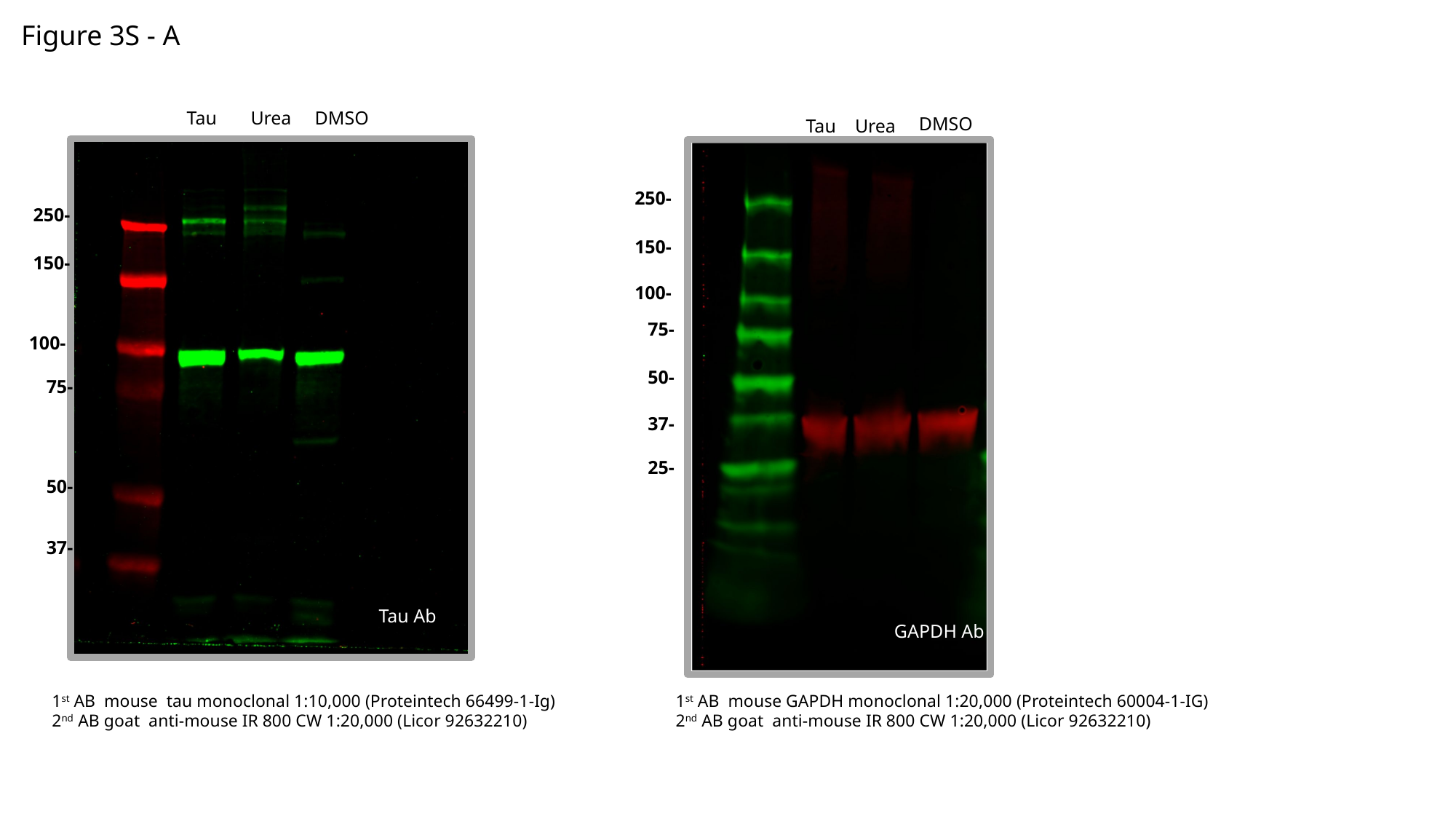

Figure 3S - A
Tau
Urea
DMSO
DMSO
Tau
Urea
250-
150-
100-
 75-
 50-
 37-
 25-
250-
150-
100-
 75-
 50-
 37-
Tau Ab
GAPDH Ab
1st AB mouse GAPDH monoclonal 1:20,000 (Proteintech 60004-1-IG)
2nd AB goat anti-mouse IR 800 CW 1:20,000 (Licor 92632210)
1st AB mouse tau monoclonal 1:10,000 (Proteintech 66499-1-Ig)
2nd AB goat anti-mouse IR 800 CW 1:20,000 (Licor 92632210)

## Slide 2
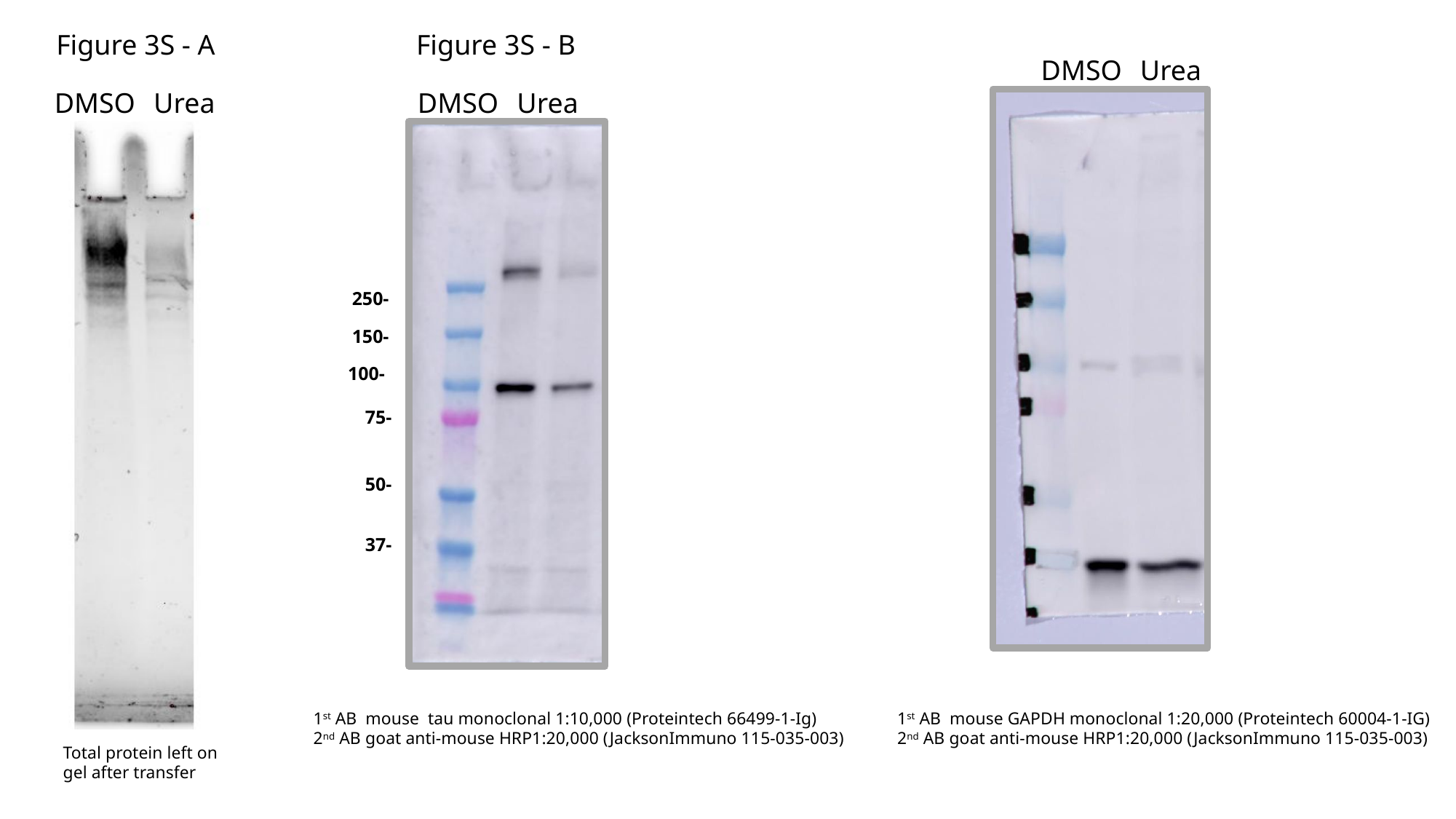

Figure 3S - A
Figure 3S - B
DMSO
Urea
DMSO
Urea
DMSO
Urea
250-
150-
100-
 75-
 50-
 37-
1st AB mouse tau monoclonal 1:10,000 (Proteintech 66499-1-Ig)
2nd AB goat anti-mouse HRP1:20,000 (JacksonImmuno 115-035-003)
1st AB mouse GAPDH monoclonal 1:20,000 (Proteintech 60004-1-IG)
2nd AB goat anti-mouse HRP1:20,000 (JacksonImmuno 115-035-003)
Total protein left on gel after transfer

## Slide 3
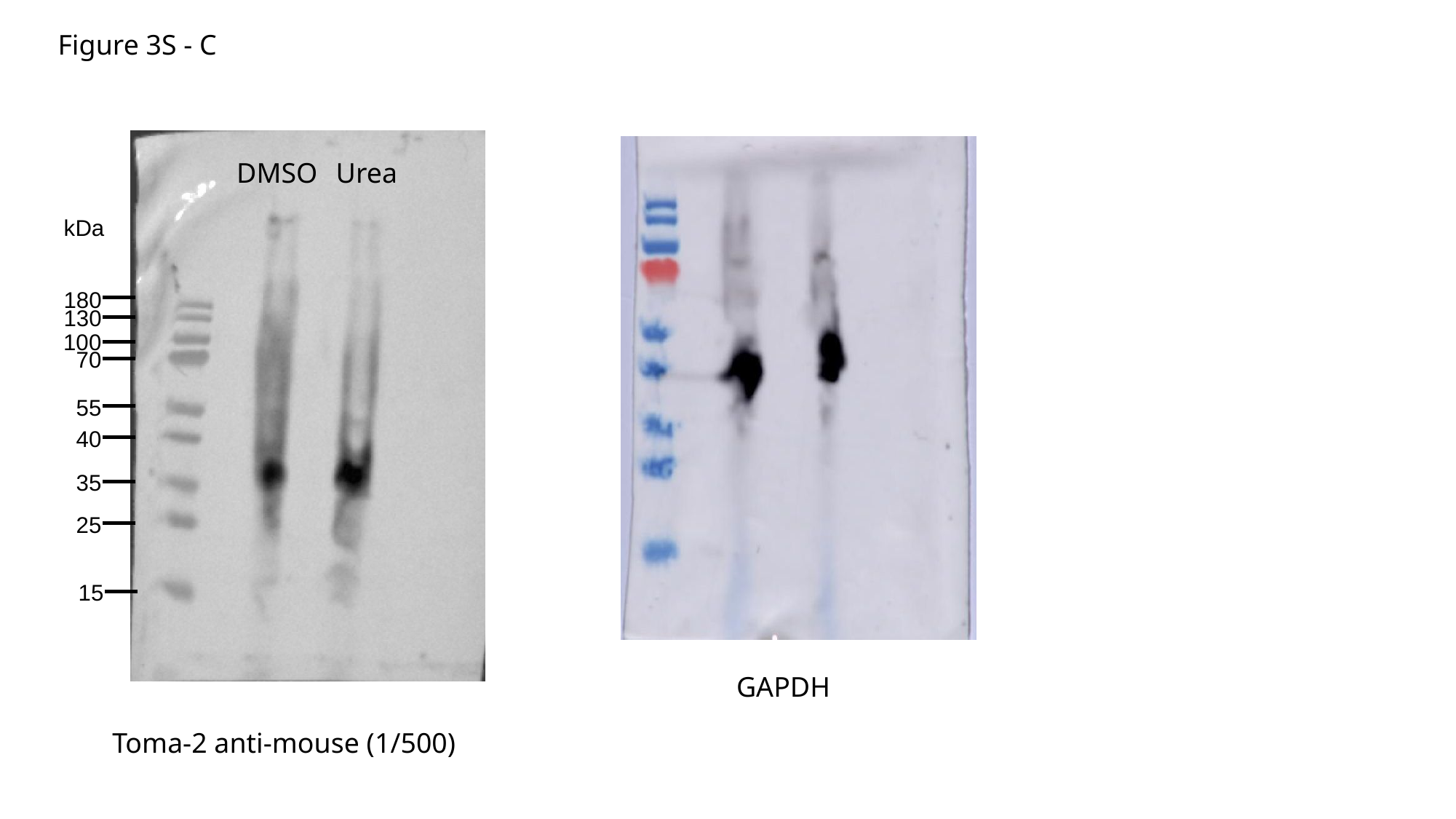

Figure 3S - C
DMSO
Urea
kDa
180
130
100
70
55
40
35
25
15
GAPDH
Toma-2 anti-mouse (1/500)

## Slide 4
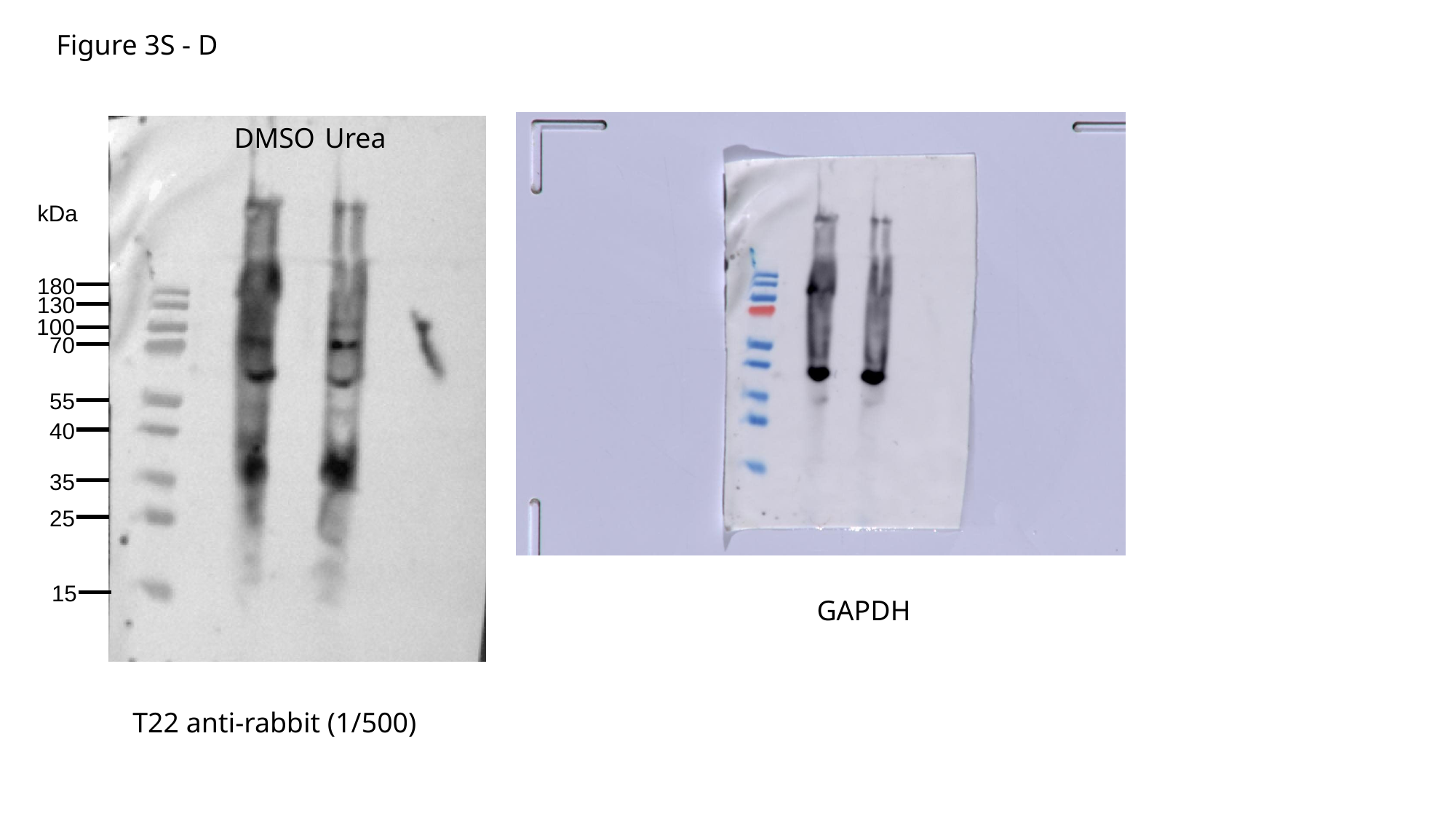

Figure 3S - D
DMSO
Urea
kDa
180
130
100
70
55
40
35
25
15
GAPDH
T22 anti-rabbit (1/500)

## Slide 5
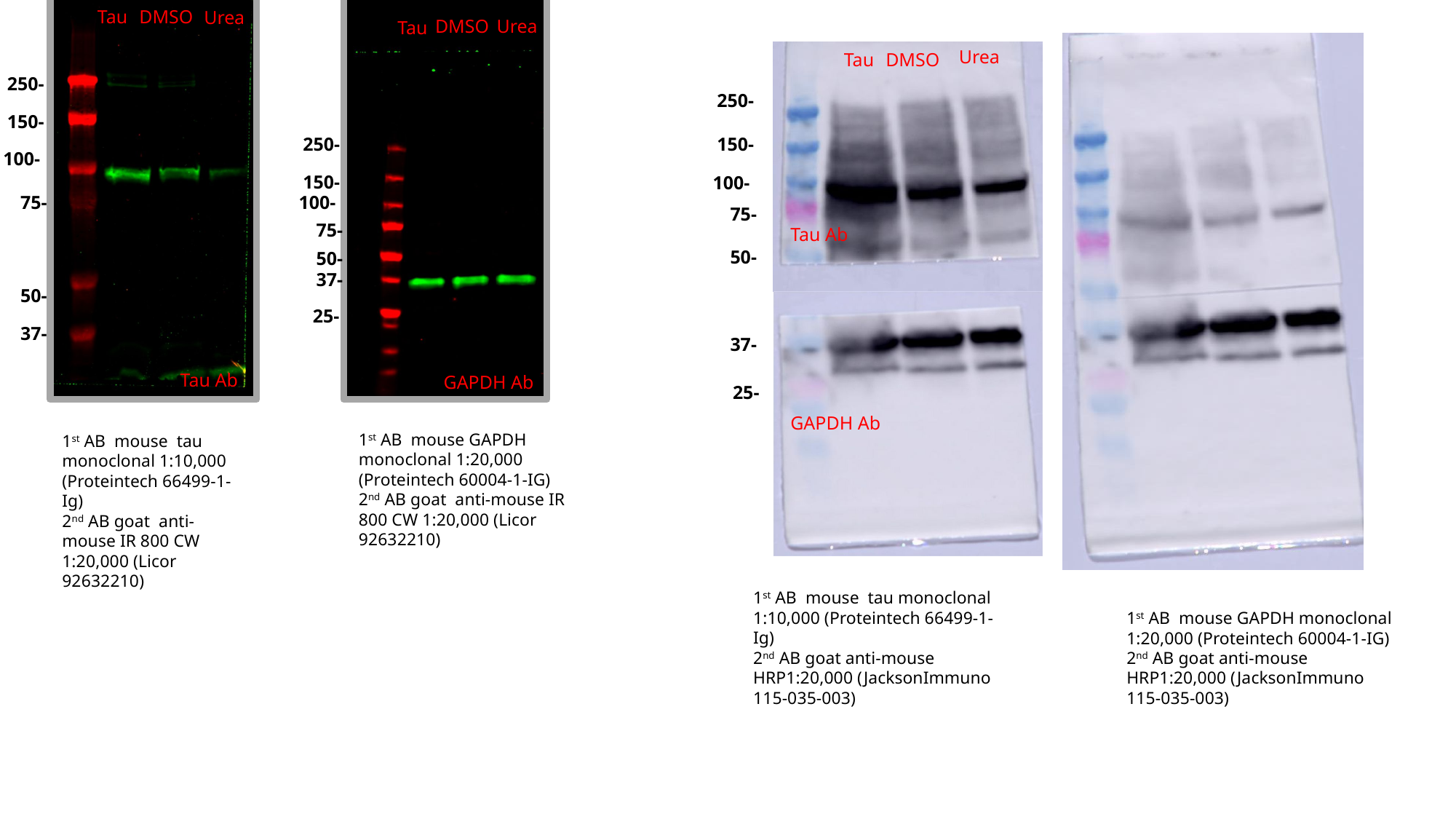

DMSO
Tau
Tau Ab
Urea
Urea
DMSO
Tau
Urea
DMSO
Tau
250-
150-
100-
 75-
 50-
 37-
250-
150-
100-
 75-
 50-
 37-
250-
150-
100-
 75-
 50-
 37-
Tau Ab
 25-
GAPDH Ab
 25-
GAPDH Ab
1st AB mouse GAPDH monoclonal 1:20,000 (Proteintech 60004-1-IG)
2nd AB goat anti-mouse IR 800 CW 1:20,000 (Licor 92632210)
1st AB mouse tau monoclonal 1:10,000 (Proteintech 66499-1-Ig)
2nd AB goat anti-mouse IR 800 CW 1:20,000 (Licor 92632210)
1st AB mouse tau monoclonal 1:10,000 (Proteintech 66499-1-Ig)
2nd AB goat anti-mouse HRP1:20,000 (JacksonImmuno 115-035-003)
1st AB mouse GAPDH monoclonal 1:20,000 (Proteintech 60004-1-IG)
2nd AB goat anti-mouse HRP1:20,000 (JacksonImmuno 115-035-003)

## Slide 6
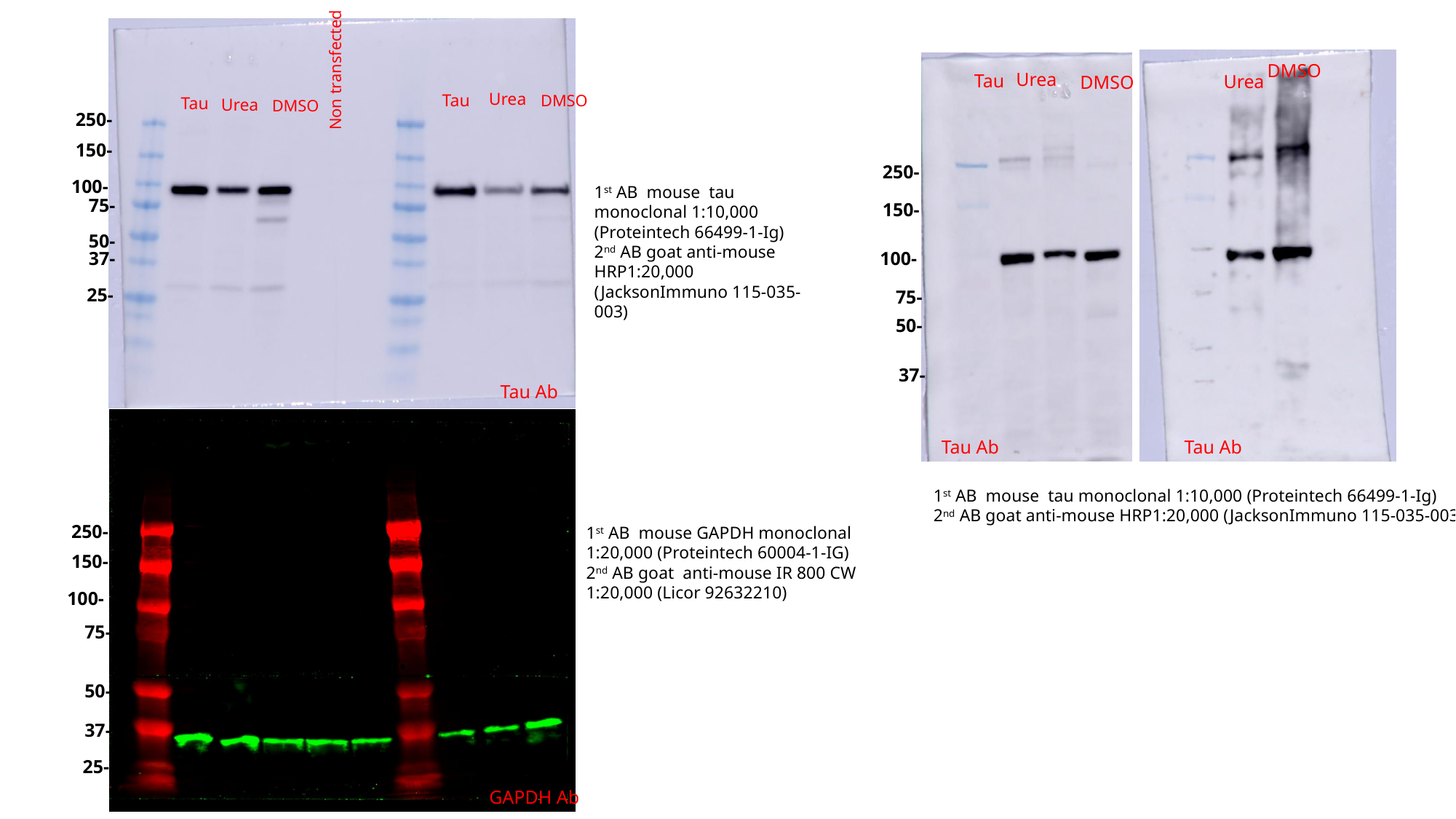

Non transfected
DMSO
Urea
Tau
Urea
DMSO
250-
150-
100-
 75-
 50-
 37-
Tau Ab
Tau Ab
1st AB mouse tau monoclonal 1:10,000 (Proteintech 66499-1-Ig)
2nd AB goat anti-mouse HRP1:20,000 (JacksonImmuno 115-035-003)
Urea
Tau
DMSO
Tau
Urea
DMSO
250-
150-
100-
 75-
 50-
 37-
1st AB mouse tau monoclonal 1:10,000 (Proteintech 66499-1-Ig)
2nd AB goat anti-mouse HRP1:20,000 (JacksonImmuno 115-035-003)
 25-
Tau Ab
250-
150-
100-
 75-
 50-
 37-
1st AB mouse GAPDH monoclonal 1:20,000 (Proteintech 60004-1-IG)
2nd AB goat anti-mouse IR 800 CW 1:20,000 (Licor 92632210)
 25-
GAPDH Ab
